# Dysfunction of the Glymphatic System Might Be Related to Iron Deposition in the Normal Aging Brain

**DOI:** 10.3389/fnagi.2020.559603

**Published:** 2020-12-21

**Authors:** Wei Zhou, Bo Shen, Wei-qiang Shen, Hao Chen, Yi-feng Zheng, Jing-jing Fei

**Affiliations:** ^1^Department of Radiology, Huzhou Central Hospital, Affiliated Central Hospital of Huzhou University, Affiliated Huzhou Hospital, Zhejiang University School of Medicine, Huzhou, China; ^2^Department of Internal Medicine, Huzhou Central Hospital, Affiliated Central Hospital of Huzhou University, Affiliated Huzhou Hospital, Zhejiang University School of Medicine, Huzhou, China

**Keywords:** glymphatic system, iron deposition, aging, brain, MRI

## Abstract

**Objective:** The study aims to detect the potential relationship between iron deposition and the function of the glymphatic system in the normal aging brain.

**Methods:** We recruited 213 healthy participants. We evaluated the function of the glymphatic system using the index for diffusivity along the perivascular space (ALPS-index), assessed iron deposition on quantitative susceptibility mapping (QSM), and analyzed their relationship.

**Results:** The mean age of participants was 60.1 ± 7.3, and 107 (50.2%) were female. The mean ALPS-index was 1.4 ± 0.2. The QSM values of the caudate nucleus, putamen, globus pallidus, thalamus, red nucleus, substantia nigra, and dentate nucleus were all related to the ALPS-index (all *P* < 0.001).

**Conclusions:** The main finding of the current study is that the regional brain iron deposition was related to the function of the glymphatic system.

**Advances in knowledge:** We first evaluated the relationship between deposition of brain iron and the dysfunction of the glymphatic system.

## Introduction

Iron is an electron facilitator and is involved in many brain functions, including oxygen transport, myelin production, electron transfer, and neurotransmitter synthesis (Hare et al., 2013). Both imaging and postmortem analyses have shown that the concentration of iron in the brain is not uniform. Previous studies have demonstrated that iron accumulates in the normal aging brain, which might damage precognitive function (Ramos et al., 2014; Gong et al., 2015). However, the exact mechanism of iron deposition in the aging brain remains unclear.

Recent work has led to the discovery of the “glymphatic system,” which is a coined phrase that combines “gl” for glia cell with “lymphatic system” (Plog and Nedergaard, 2018; Rasmussen et al., 2018). Within the glymphatic system, cerebrospinal fluid enters the brain via peri-arterial spaces, passes into the interstitium via astrocytic aquaporin-4, and then drives the peri-venous drainage of interstitial fluid and its solute (Plog and Nedergaard, 2018; Rasmussen et al., 2018). Evidence suggests that the glymphatic system is an important fluid-clearance system in the brain (Bakker et al., 2016). Numerous neurological disorders have been found to be closely related to the dysfunction of the glymphatic system, including Alzheimer's disease and Parkinson's disease (Rasmussen et al., 2018; Zou et al., 2019). Evidence also revealed that iron deposition was one of the most important underlying mechanisms in Alzheimer's disease and Parkinson's disease (Liu et al., 2018; Chen et al., 2019). Some scholars also believe that the glymphatic system may be the major contributory factor to the deposition and clearance of iron in brain tissue (Wang et al., 2020), but evidence is still lacking.

Taoka et al. proposed a new method using diffusion tensor imaging analysis along the perivascular space (ALPS)-index to evaluate the glymphatic system using diffusion tensor imaging (DTI) and susceptibility weighted imaging (SWI) (Taoka et al., 2017). The authors evaluated water diffusivity along the right-to-left direction of the periventricular brain tissue, which matched the running direction of the deep medullary veins. Expected water diffusivity along the right-to-left direction could partially reflect the function of the glymphatic system. Using this method, they demonstrated a significant correlation between water diffusivity along the right-to-left direction and severity of cognitive dysfunction in Alzheimer's disease.

Therefore, in this study, we evaluated the function of the glymphatic system by ALPS-index based on DTI and SWI, assessed brain regional iron deposition using quantitative susceptibility mapping (QSM) based on SWI, and analyzed the relationship between them, aiming to discover the link between the glymphatic system and iron deposition in brain tissues.

## Materials and Methods

### Patients

This study was approved by our institutional review board, and the requirement for patient consent was waived. From December 2017 to September 2019, 213 healthy participants (106 male and 107 female, age range 43–80) underwent multi-mode MRI scans.

### MRI Protocol

MRI was performed on a 3.T scanner (GE Discovery MR750). The standard MRI protocol included axial unenhanced T1-weighted imaging (T1WI) (TR, 500 ms; TE, 10 ms; slice thickness, 5 mm; field of view, 240 mm; matrix = 320 × 356), DTI (b, 0; and b, 2,000 s/mm^2^, TR, 6,600 ms, TE, 89 ms, MPG, 30 directions, field of view, 230 mm, matrix = 94 × 94, slice thickness, 3 mm), and SWI (TR, 22 ms; TE, 11.5 ms; field of view, 230 mm; slice thickness, 2 mm; matrix, 320 × 251; flip angle, 20°).

### Image Analysis

**Figure 1:**
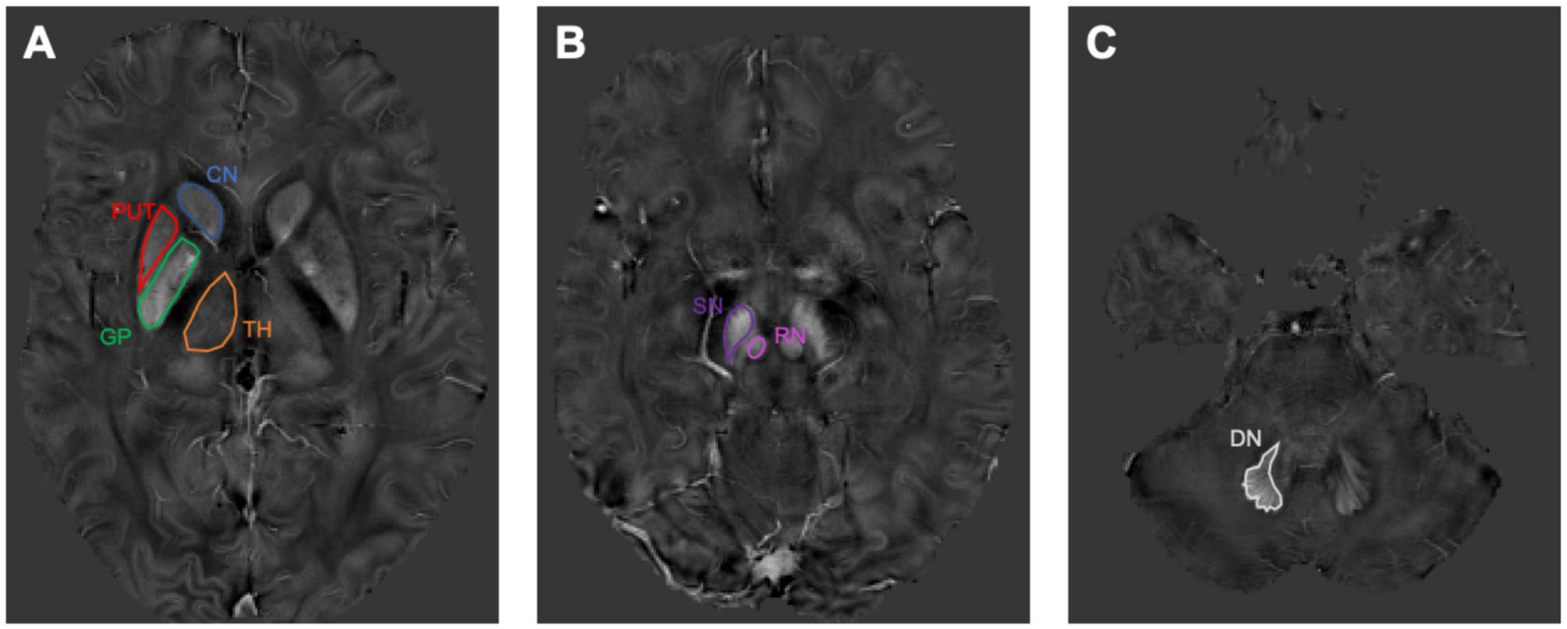
The susceptibility maps illustrate the selected regions of interest covering the cerebrum, midbrain, and cerebellum. **(A)** shows the region of interests (ROIs) of caudate nucleus (CN), putamen (PUT), globus pallidus (GP), and thalamus (TH). **(B)** shows the ROIs of red nucleus (RN) and substantia nigra (SN). **(C)** shows the ROI of dentate nucleus (DN).

The magnitude image and phase image were generated from the SWI raw image. Then the magnitude image was processed to generate a brain mask on a brain extraction tool contained in the FMRIB Software Library (Jenkinson et al., 2012). Phase images were divided by 2πT_E_ to obtain a raw frequency map, and then the background field frequency was removed using the modified SHARP method (Schweser et al., 2011). Then the QSM mapping was derived by the LSQR method (Li et al., 2011). The regions of interest (ROIs) were determined by a previous publication (Guan et al., 2017). ITK-SNAP (www.itksnap.org) was used to perform manual segmentation and to measure the QSM values for each nucleus. Data for each region were obtained from the entire visible slice. ROIs were drawn to cover the caudate nucleus, putamen, globus pallidus, thalamus, red nucleus, substantia nigra, and dentate nucleus (Figure 1). An experienced neuroradiologist responsible for the ROI analysis was blinded to the information about the subjects, including disease status and demographics.

The ALPS-index was calculated as previously described by Taoka et al. (2017). On the color-coded fractional anisotropy map of the plane at the level of the lateral ventricle, 5-mm-diameter circle ROIs in the area of the projection fibers and in the area of the association fibers in the left hemisphere were performed by an experienced radiologist, who was blinded to the clinical data and other images. Then ALPS-index was calculated as ALPS index = mean (D*xproj* + D*xassoc*)/mean (D*yproj* + D*zassoc*).

We auto-segmented white matter hyperintensities using the LST toolbox and measured the volumes of white matter hyperintensities (Wirth et al., 2019). Central and cortical brain atrophy were categorized into three scores (0 = none, 1 = modest, and 2 = severe) as shown in a previous study (Sato et al., 2016).

### Statistical Analysis

Statistical analyses were performed using the SPSS software version 22. All metric and normally distributed variables were reported as mean ± standard deviation. Categorical variables were presented as frequency (percentage). Correlate associations between variables were assessed by Spearman correlation analysis. Differences were considered statistically significant at *P* < 0.05. Since the values of seven nucleus were measured, the statistically significant *P*-value for correlate associations of nucleus and ALPS-index should be <0.007, according to the basic tests of Bonferroni.

## Results

**Figure 2:**
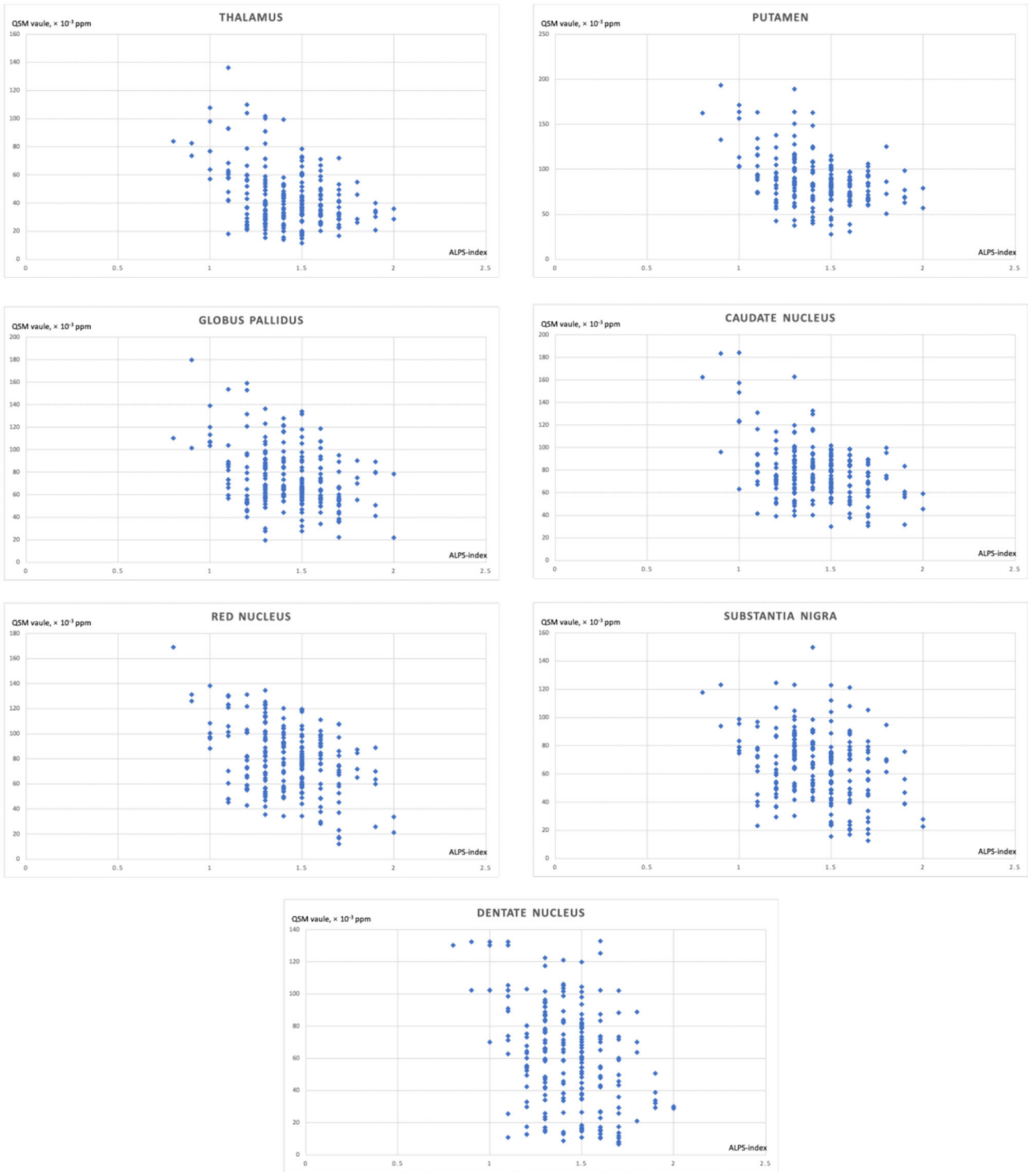
The correlation between the index for diffusivity along the perivascular space (ALPS-index) and regional brain iron depositions.

In this study, 213 patients were included. Table 1 shows the demographic data. The mean age was 60.1 ± 7.3, and 107 (50.2%) were female. The mean ALPS-index was 1.4 ± 0.2. The mean volumes of white matter hyperintensities were 9.7 ± 6.8. The median scores of brain atrophy on the cortical and center were 1 (1, 2) and 1 (1, 2), respectively. Table 2 shows the relationship between the ALPS-index and regional brain iron depositions. The QSM values of the caudate nucleus, putamen, globus pallidus, thalamus, red nucleus, substantia nigra, and dentate nucleus were all related to the ALPS-index (all *P* < 0.05). Moreover, age was related to both the ALPS-index (*r* = −0.263, *P* < 0.001) and regional QSM values (caudate nucleus: *r* = 0.218, *P* = 0.001; putamen: *r* = 0.373, *P* < 0.001; globus pallidus: *r* = 0.180, *P* = 0.009; thalamus: *r* = 0.164, *P* = 0.016; red nucleus: *r* = 0.182, *P* = 0.008; substantia nigra: *r* = 0.199, *P* = 0.004; dentate nucleus: *r* = 0.213, *P* = 0.002). Table 3 shows the relationship between ALPS-index and regional brain iron depositions after adjusting for age, volumes of white matter hyperintensities, and scores of brain atrophy. Figure 2 shows the correlation between the index for diffusivity along the perivascular space (ALPS-index) and regional brain iron depositions.

**Table 1:** Demographic and basic imaging data.

**Variables**	**Mean ± SD**
Age, year	60.1 ± 7.3
ALPS-index	1.4 ± 0.2
Caudate nucleus, × 10^−3^ ppm	79 ± 25
Putamen, × 10^−3^ ppm	88 ± 28
Globus pallidus, × 10^−3^ ppm	75 ± 27
Thalamus, × 10^−3^ ppm	45 ± 22
Red nucleus, × 10^−3^ ppm	79 ± 27
Substantia nigra, × 10^−3^ ppm	66 ± 24
Dentate nucleus, × 10^−3^ ppm	61 ± 32

*ALPS-index: the index for diffusivity along the perivascular space*.

**Table 2:** The relationship between the index for diffusivity along the perivascular space (ALPS-index) and regional brain iron depositions.

	**ALPS-index (r, P)**
Caudate nucleus, × 10^−3^ ppm	−0.289, <0.001
Putamen, × 10^−3^ ppm	−0.337, <0.001
Globus pallidus, × 10^−3^ ppm	−0.280, <0.001
Thalamus, × 10^−3^ ppm	−0.284, <0.001
Red nucleus, × 10^−3^ ppm	−0.307, <0.001
Substantia nigra, × 10^−3^ ppm	−0.267, <0.001
Dentate nucleus, × 10^−3^ ppm	−0.333, <0.001

**Table 3:** Multiple factors analysis of the index for diffusivity along the perivascular space (ALPS-index).

	**Age (β, P) (adjusting for ALPS-index)**	**ALPS-index (β, P) (adjusting for age)**	**ALPS-index (β, P) (adjusting for age, volumes of white matter hyperintensities and scores of brain atrophy)**
Caudate nucleus, × 10^−3^ ppm	−0.175, 0.006	−0.374, <0.001	−0.380, <0.001
Putamen, × 10^−3^ ppm	−0.146, 0.028	−0.354, <0.001	−0.342, <0.001
Globus pallidus, × 10^−3^ ppm	−0.198, 0.003	−0.301, <0.001	−0.306, <0.001
Thalamus, × 10^−3^ ppm	−0.200, 0.002	−0.339, <0.001	−0.355, <0.001
Red nucleus, × 10^−3^ ppm	−0.185, 0.004	−0.359, <0.001	−0.372, <0.001
Substantia nigra, × 10^−3^ ppm	−0.213, 0.001	−0.254, <0.001	−0.260, <0.001
Dentate nucleus, × 10^−3^ ppm	−0.200, 0.002	−0.343, <0.001	−0.361, <0.001

## Discussion

The main finding of the current study is that regional iron deposition in brain tissues was related to the function of the glymphatic system in normal aging persons. Previously, the glymphatic system has been speculated to be responsible for the clearance and homeostasis of waste in the brain (Bakker et al., 2016; Plog and Nedergaard, 2018; Rasmussen et al., 2018), but it remains unknown whether iron metabolism was related to the glymphatic system. Most recently, in animal models of intraventricular hemorrhage, the ability of iron drainage through deep cervical lymph nodes (DCLNs) was confirmed by Perl's Prussian blue reaction. They found that the DCLNs-excised group showed higher ferritin levels at areas surrounding ventricles than a DCLNs-preserved group 3 days after intraventricular hemorrhage (Wang et al., 2020). The result indicated the possible relationship between the function of the glymphatic system and iron drainage, since the DCLNs were recognized as downstream of the glymphatic system (Eide et al., 2018; Zhou et al., 2020). Our results support that in a healthy aging brain, the glymphatic system might also be involved in the clearance of iron, suggesting that iron metabolism shared the same pathway as other waste metabolisms. Moreover, a study has demonstrated that injury of the microvasculature and capillary-level microhemorrhages coincided with amyloid beta (Aβ) deposits in senile plaques (Hansra et al., 2019). Iron deposition plays an important role in cerebral small vessel diseases (Del C Valdés Hernández et al., 2015). Therefore, we inferred that dysfunction of the glymphatic system might lead to the damage of microvasculature via deposition of Aβ, then leading to iron deposition.

Moreover, iron deposition might also lead to the dysfunction of the glymphatic system. Iron metabolism mainly depends on iron regulatory proteins including ferritin, transferrin and transferrin receptor, hepcidin, ferroportin, and lactoferrin. A previous study had demonstrated that abnormal iron metabolism could generate hydroxyl radicals via the Fenton reaction, which could further trigger oxidative stress reactions, damage cell lipids, protein, and DNA structure and function, lead to cell death, and ultimately influence the process of Aβ misfolding and plaque aggregation (Wang et al., 2019). The aggregation and deposition of Aβ in perivascular space may affect the bulk flow in perivascular space, directly leading to the dysfunction of the glymphatic system (Mestre et al., 2017). In addition, both iron deposition and dysfunction of the glymphatic system have been implicated in the pathogenesis of Alzheimer's Disease, Parkinson's Disease, and secondary injury following intracerebral hemorrhage, which supports the relationship between iron deposition and glymphatic function (Rasmussen et al., 2018; Farr and Xiong, 2020).

We also found a negative relationship between the function of the glymphatic system and age, which is in accordance with previous studies (Kress et al., 2014; Del C Valdés Hernández et al., 2015). Moreover, when both age and ALPS-index were set as independent variables, they were both independently related with regional QSM values. It might suggest that, although there are similar tends in increasing of iron deposition and decreasing of glymphatic function with age, both of them have an independent effect on glymphatic dysfunction. Further studies are needed to clarify the mechanism.

An advantage of this study is that it is the first evaluation of the relationship between chronic deposition of iron and dysfunction of the glymphatic system. Our study also has several limitations. First, the subjects were restricted in our single center. Further multiple center studies with larger samples are needed. Second, the ROIs were placed manually, which may be a subjective factor of our measurement, although the radiologist is experienced and was blinded to clinical data and other images. Third, the method of the ALPS-index is theoretically deductive. Although it has demonstrated a relation with the severity of Alzheimer's disease and idiopathic normal pressure hydrocephalus, histological verification has not been performed.

## Conclusion

The main finding of the current study is that regional brain iron deposition was related to the function of the glymphatic system.

## Data Availability Statement

The original contributions presented in the study are included in the article/supplementary material, further inquiries can be directed to the corresponding author/s.

## Ethics Statement

The studies involving human participants were reviewed and approved by Huzhou Central Hospital Ethics Committee. The patients/participants provided their written informed consent to participate in this study.

## Author Contributions

BS designed the experiments. WZ carried out experiments. W-qS and HC analyzed experimental results. Y-fZ and J-jF participated in data collection. All authors wrote the manuscript.

## Conflict of Interest

The authors declare that the research was conducted in the absence of any commercial or financial relationships that could be construed as a potential conflict of interest.
